# Foliar Application of MgO Nanoparticles Modulates Magnesium Nutrition and Fruit Quality in Loquat Under Mg-Deficient Conditions

**DOI:** 10.3390/plants15132099

**Published:** 2026-07-06

**Authors:** Yuxiao Yang, Jinrun Ni, Wenkai Wang, Chang Lu, Jingjing Wan, Bilal Hussain, Xiaoe Yang, Shane Wang

**Affiliations:** State Key Laboratory of Soil Pollution Control and Safety, Zhejiang University, Hangzhou 310058, China

**Keywords:** loquat, magnesium oxide nanoparticles, foliar application, fruit quality, sugar–acid metabolism

## Abstract

Magnesium (Mg) deficiency is common in acidic orchard soils and can limit fruit crop growth and quality. This study evaluated whether foliar-applied magnesium oxide nanoparticles (MgO NPs) could improve Mg nutrition and fruit quality in ‘Ninghaibai’ loquat grown under Mg-deficient acidic soil conditions. Pot and field experiments were conducted using water as the control and MgSO_4_-50eq as an equimolar Mg comparator. MgO NPs showed a concentration-dependent effect, and 200 mg/L produced the best overall performance among the tested concentrations. At this concentration, total biomass increased by 47.27%, compared with CK, accompanied by enhanced chlorophyll accumulation, antioxidant enzyme activities, and Mg uptake. In fruit, 200 mg/L MgO NPs increased soluble solids content by 45.67% and reduced titratable acidity by 53.26%, while also improving fruit size and sugar–acid balance. Leaf transcriptome analysis suggested that MgO NPs altered the expression of genes involved in metabolism, stress response, and secondary metabolite biosynthesis. At the 50 mg/L level, MgO NPs produced stronger responses than the equimolar MgSO_4_ treatment in Mg uptake, nutrient acquisition, and several fruit-quality traits. However, excessive application at 500 mg/L weakened growth and quality improvement. Overall, foliar application of 200 mg/L MgO NPs may represent a promising strategy for improving loquat growth and fruit quality under the tested Mg-deficient conditions.

## 1. Introduction

Loquat (*Eriobotrya japonica* Lindl.) is an economically important subtropical fruit crop that is widely appreciated for its distinctive flavor, tender texture and high market value [1]. However, the stable production of high-quality loquat fruit is often hindered by factors such as weak seedling growth, low nutrient use efficiency, mineral nutrient deficiencies in orchard soils, and inconsistent fruit quality. These issues can reduce tree vigor, restrict nutrient accumulation and ultimately impact fruit size, sugar–acid balance, nutritional composition and postharvest commercial quality [1,2,3,4]. Therefore, improving nutrient management is essential for enhancing both vegetative growth and fruit quality in loquat production [5,6,7].

Magnesium (Mg) is an essential macronutrient for plant growth and development. It is the central atom of the chlorophyll molecule and is directly involved in photosynthesis, carbon fixation, carbohydrate transport, enzyme activation, and energy metabolism. Adequate Mg supply can improve chlorophyll synthesis, photosynthetic efficiency, antioxidant capacity, and biomass accumulation [5,8,9]. In addition, Mg plays an important role in nutrient interaction and transport, particularly in the uptake, assimilation, and redistribution of nitrogen, phosphorus, and potassium [5,10]. Mg deficiency often leads to impaired photosynthesis, excessive reactive oxygen species accumulation, reduced carbohydrate transport, and abnormal fruit development [8,9]. In fruit crops, Mg nutrition is closely associated with fruit yield and quality, including fruit size, soluble solids, organic acid metabolism, sugar accumulation, and the biosynthesis of antioxidant compounds [7,11]. However, conventional Mg fertilizers may show limited efficiency because of nutrient leaching, soil fixation, poor mobility, and low foliar absorption efficiency under certain cultivation conditions [10,12]. Thus, developing more efficient Mg fertilization strategies is important for high-quality fruit production.

Nano fertilizers have attracted increasing attention as potential tools for improving nutrient-use efficiency and crop performance [12,13,14]. Owing to their small particle size, large specific surface area, and unique physicochemical properties, nanoparticles may improve nutrient delivery, enhance foliar retention, and regulate plant physiological responses at relatively low application rates [12,13,15,16,17]. Among Mg-based nanomaterials, magnesium oxide nanoparticles (MgO NPs) have shown potential in promoting plant growth, improving stress tolerance, and regulating nutrient metabolism in several crop species [18,19,20]. Compared with conventional soluble Mg fertilizers, MgO NPs may provide a more efficient Mg source through gradual nutrient release, improved leaf surface interaction, and enhanced physiological activity [15,16,18]. Nevertheless, plant responses to nanoparticles are highly dependent on crop species, nanoparticle properties, application method, and dosage [12,15,16,19]. Low or moderate concentrations may promote growth and metabolism, whereas excessive concentrations may inhibit plant development or induce stress responses [19,20]. However, nano-sized metal oxide fertilizers may also pose risks related to nanoparticle toxicity, environmental persistence, and potential effects on soil health and non-target organisms [21]. Therefore, determining the appropriate application concentration is critical for the safe and effective use of MgO NPs in fruit crop production.

Although MgO NPs have been investigated in some agricultural systems, their effects on loquat remain poorly understood. In particular, limited information is available on whether foliar-applied MgO NPs can provide additional benefits beyond conventional soluble Mg supplementation in improving loquat growth, nutrient uptake, and fruit quality. Moreover, the physiological and metabolic mechanisms underlying MgO NP-induced quality improvement in loquat have not been systematically clarified. Fruit quality formation is a complex process involving nutrient supply, photosynthetic carbon assimilation, antioxidant metabolism, sugar–acid balance, and secondary metabolite biosynthesis [7,11,22,23,24]. Since leaves are the primary source organs for photosynthate production and nutrient redistribution, changes in leaf physiological and metabolic status may influence fruit development and quality formation [8,9,11]. Therefore, integrating growth, nutrient uptake, fruit quality, and transcriptomic analyses can provide a more comprehensive understanding of the response of loquat to MgO NP application [18,22,24].

In this study, ‘Ninghaibai’ loquat was used as the experimental material to evaluate the effects of foliar application of MgO NPs on seedling growth, nutrient absorption, antioxidant enzyme activities in the pot experiment, as well as fruit quality in mature fruiting trees under field conditions. Water treatment was used as the control, and MgSO_4_-50eq was used as the conventional Mg fertilizer comparator. Pot and field experiments were conducted to determine the optimal MgO NP concentration and to compare MgO NPs with MgSO_4_ under the tested equimolar Mg condition. In addition, transcriptomic analyses of loquat leaves were performed to explore potential pathways associated with MgO NP-induced changes in plant metabolism and fruit quality formation. We hypothesized that foliar MgO NPs could improve loquat growth and fruit quality by enhancing Mg nutrition, promoting macronutrient uptake, improving antioxidant-related physiological responses, and regulating secondary metabolism. Unlike previous studies that mainly focused on conventional Mg fertilization or single agronomic responses, this study evaluates the concentration-dependent effects of MgO NPs and compares MgO NPs with MgSO_4_-50eq under an equimolar Mg condition, while integrating pot and field experiments with transcriptomic analysis in loquat. The results of this study provide theoretical and practical evidence for the potential application of MgO NPs as an efficient nano-magnesium fertilizer in high-quality loquat cultivation.

## 2. Results and Discussion

### 2.1. Foliar MgO NPs Improved Loquat Seedling Growth, Photosynthetic Pigment Accumulation, and Antioxidant Enzyme Activities

The experimental soil was severely deficient in exchangeable Mg (7.19 mg/kg; Table S1), providing a clear background for evaluating the physiological effect of foliar Mg supplementation. MgO NPs showed a concentration-dependent promotion of seedling growth (Table S2). Compared with CK, MgO NPs at 50–300 mg/L significantly increased root and above-ground biomass, whereas the 500 mg/L treatment weakened the growth-promoting effect and reduced root biomass to a level not significantly different from CK (Table S2). At 200 mg/L MgO NPs, root and total biomass reached their highest values of 1.39 and 5.16 g/plant DW, respectively, representing increases of 72.12% and 47.27% over CK (Table S2). In contrast, above-ground, stem, and leaf biomass reached their highest values at 300 mg/L MgO NPs (Table S2). At the equimolar Mg level, MgO NPs-50 increased root and above-ground biomass by 17.73% and 10.67%, respectively, compared with MgSO_4_-50eq (Table S2), indicating that MgO NPs-50 was more effective than MgSO_4_-50eq in promoting early vegetative growth.

**Figure 1:**
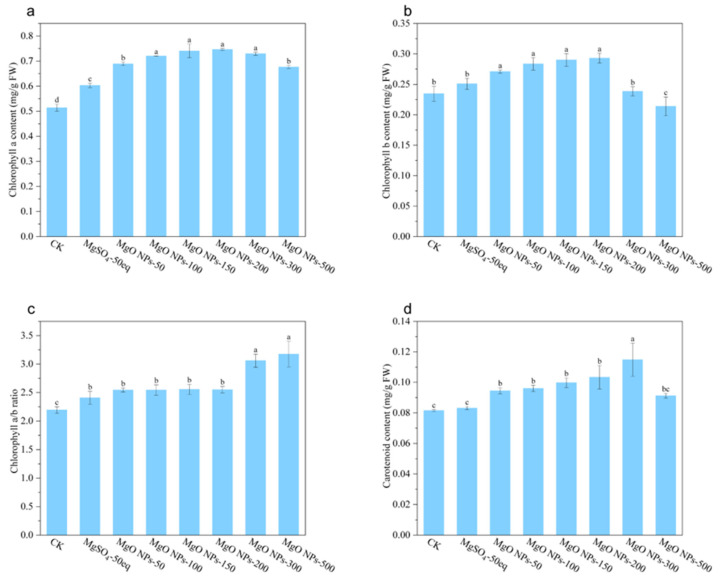
Effects of MgO NPs on photosynthetic pigment-related parameters in loquat seedling leaves: (**a**) chlorophyll a content, (**b**) chlorophyll b content, (**c**) chlorophyll a/b ratio, and (**d**) carotenoid content. Different lowercase letters above the bars indicate significant differences among different treatments (*p* < 0.05). Data are presented as mean ± SD, *n* = 3. FW stands for fresh weight.

The improvement in seedling growth was closely associated with enhanced photosynthetic pigment accumulation. Chlorophyll a and chlorophyll b contents peaked at 200 mg/L MgO NPs, reaching 0.75 and 0.29 mg/g FW and increasing by 45.25% and 24.92% over CK (Figure 1a,b). The chlorophyll a/b ratio also increased under suitable MgO NP concentrations (Figure 1c). Carotenoid content continued to increase up to 300 mg/L MgO NPs, where it reached 0.11 mg/g FW, 40.81% higher than CK, before declining markedly at 500 mg/L (Figure 1d). At the equimolar Mg level, MgO NPs-50 produced stronger pigment responses than MgSO_4_-50eq, increasing chlorophyll a, chlorophyll b, and carotenoid contents by 14.30%, 8.03%, and 13.65%, respectively (Figure 1a,b,d). This pattern suggests that moderate MgO NP application improves chlorophyll synthesis and light-harvesting capacity, whereas the pigment-promoting effect declined at 500 mg/L MgO NPs. Because Mg is the central atom of chlorophyll and is essential for photosynthetic carbon fixation and assimilate transport [8,22], the pigment response provides a physiological basis for the observed biomass increase.

MgO NPs also enhanced the antioxidant enzyme activities of loquat seedling leaves (Table S3). Relative to MgSO_4_-50eq, MgO NPs-50 significantly increased SOD, POD, and CAT activities and soluble protein content by 22.56%, 19.02%, 10.37%, and 10.44%, respectively (Table S3). The highest SOD, POD, and CAT activities were recorded at 300 mg/L MgO NPs (158.73, 1198.80, and 6.41 U/g FW), corresponding to increases of 59.55%, 42.72%, and 23.52% compared with CK (Table S3). However, soluble protein content was already slightly lower at 300 mg/L than at 150–200 mg/L, and all antioxidant-related indicators declined at 500 mg/L (Table S3). These results indicate that MgO NPs can enhance enzymatic antioxidant responses at suitable concentrations, whereas excessive application may weaken these responses and impair seedling physiological performance. Such a hormetic response has also been observed in other MgO NP-treated crops. For example, Ali et al. reported that low-dose MgO NPs, especially 10 mg/L, promoted seed germination, shoot and root growth, biomass accumulation, photosynthetic pigments, soluble protein, carbohydrate accumulation, and antioxidant defense in Brassica napus, whereas excessive MgO NPs, particularly 200 mg/L, induced oxidative stress, inhibited photosynthesis, promoted protein oxidation and carbohydrate degradation, and ultimately suppressed plant growth [19]. Similarly, Taj et al. found that 200 ppm MgO NPs was the optimal dose for improving spinach growth in a preliminary concentration screening, and subsequent foliar application of MgO NPs enhanced chlorophyll, carotenoid, SPAD value, water status, antioxidant enzyme activity, and phenolic accumulation under Cd stress [25]. In maize, Abbas et al. also showed that MgO NP treatments produced concentration-dependent improvements in seed germination, seedling growth, Mg accumulation, and plant development, with 500 ppm showing strong Mg uptake and translocation responses [26]. These examples support our observation that moderate MgO NP application can improve photosynthetic pigment accumulation and antioxidant enzyme responses, whereas these beneficial responses were weakened at 500 mg/L MgO NPs.

### 2.2. MgO NPs Enhanced Mg Nutrition and Promoted N, P, and K Uptake

**Figure 2:**
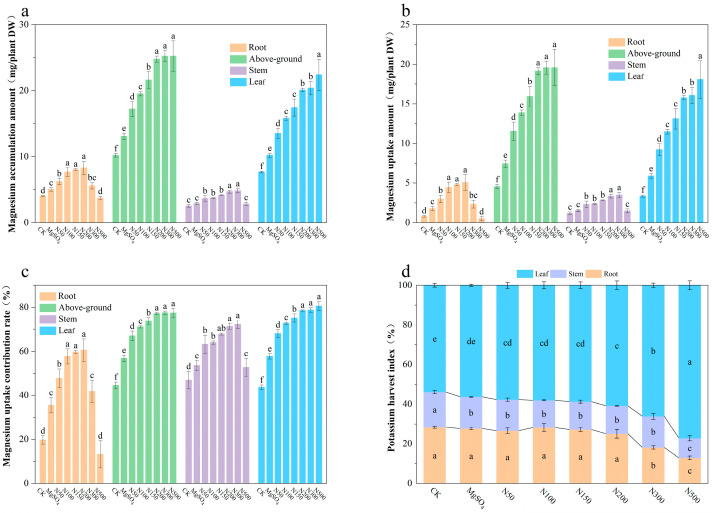
Effects of MgO NPs on (**a**) magnesium accumulation amount, (**b**) magnesium uptake amount, (**c**) magnesium uptake contribution rate, and (**d**) magnesium harvest index of loquat seedlings. N50, N100, N150, N200, N300, and N500 represent MgO NPs at 50, 100, 150, 200, 300, and 500 mg/L, respectively; MgSO_4_ represents MgSO_4_-50eq. Different lowercase letters above the bars indicate significant differences among different treatments (*p* < 0.05). Data are presented as mean ± SD, *n* = 3. DW stands for dry weight.

The Mg accumulation and uptake parameters further showed that Mg nutrition varied with the concentration of MgO NPs (Figure 2). Root Mg accumulation peaked at 200 mg/L, increasing by 105.54% compared with CK (Figure 2a), whereas root Mg uptake at this concentration increased by 534.99% (Figure 2b). At the equimolar Mg level, MgO NPs-50 significantly increased Mg accumulation in root, above-ground parts, stem, and leaves by 23.84%, 31.27%, 27.11%, and 32.44% over MgSO_4_-50eq (Figure 2a), and increased Mg uptake by 66.95%, 55.03%, 50.58%, and 56.19%, respectively (Figure 2b). These differences suggest that MgO NPs-50 produced stronger Mg accumulation and uptake responses than MgSO_4_-50eq under the tested conditions; this response may be related to nanoparticle-specific foliar retention and nutrient delivery characteristics reported in previous studies [27,28,29,30], although the detailed delivery processes remain to be further clarified.

The distribution indices showed that MgO NPs altered Mg allocation within seedlings. With increasing MgO NP concentration, the Mg harvest index of roots and stems decreased, whereas that of leaves increased from 53.89% in CK to 77.18% (Figure 2d). Mg uptake contribution also increased in root, above-ground parts, and leaves under suitable MgO NP concentrations (Figure 2c). This shift increased Mg allocation to leaves, especially at higher MgO NP concentrations. Although improved leaf Mg status may benefit photosynthesis, the reduced root Mg uptake observed at 500 mg/L indicates that the Mg-promoting effect was weakened at the highest MgO NP concentration (Figure 2b,d). Therefore, the 200 mg/L treatment appears to provide the best compromise between Mg uptake efficiency and physiological safety.

The N, P, and K results (Figures S2–S4) show that MgO NPs not only increased Mg nutrition but also improved macronutrient acquisition. Most N, P, and K accumulation and uptake parameters reached their highest values around 200 mg/L MgO NPs (Figures S2a,b–S4a,b). At this concentration, root, above-ground, and leaf N accumulation increased by 110.80%, 63.37%, and 68.24% over CK (Figure S2a), and N uptake increased by 245.79%, 302.29%, and 313.89%, respectively (Figure S2b). For P, root and leaf P uptake increased by 341.01% and 673.98% (Figure S3b), whereas for K, root, above-ground, stem, and leaf K uptake increased by 128.74%, 383.68%, 531.59%, and 314.84% (Figure S4b). Correlation analysis further showed that seedling biomass was positively associated with photosynthetic pigments, antioxidant enzyme activities, and mineral nutrient contents, especially N and K (Figure S5). These relationships suggest that the improvement in seedling vigor and biomass accumulation was associated with coordinated changes in Mg nutrition, photosynthetic pigment accumulation, antioxidant enzyme activities, and N and K nutrition. From a mechanistic perspective, the observed association between improved Mg nutrition and macronutrient uptake may be explained partly by Mg-dependent ATP utilization and H^+^-ATPase-related transport processes. Because ATP-dependent enzymes, including ATPases, generally require Mg–ATP as the effective substrate, Mg supply may help maintain the proton gradients and membrane potential generated by plasma membrane H^+^-ATPases, which drive the secondary transport of mineral nutrients such as nitrate, phosphate, and K^+^ [31,32]. Therefore, Mg supplied by MgO NPs may enhance energy-dependent nutrient transport, thereby helping to explain the observed increases in N, P, and K uptake and allocation. In addition, as reported in previous studies, the nanoscale properties of MgO NPs may improve foliar nutrient retention and delivery efficiency, thereby potentially enhancing nutrient-use efficiency [27,28,29,30,33].

### 2.3. MgO NPs Improved Fruit Physical Quality and Sugar–Acid Balance

**Figure 3:**
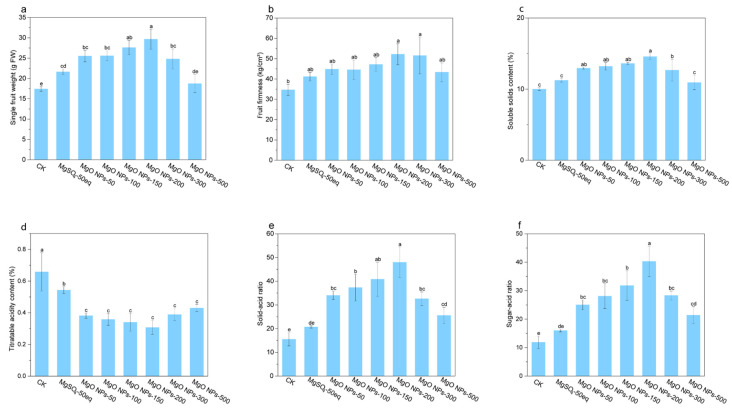
Effects of MgO NPs on the appearance quality of loquat fruit. (**a**) single fruit weight, (**b**) fruit firmness, (**c**) soluble solids content, (**d**) titratable acidity content, (**e**) solid–acid ratio and (**f**) sugar–acid ratio. Different lowercase letters above the bars indicate significant differences among different treatments (*p* < 0.05). Data are presented as mean ± SD, *n* = 3. FW stands for fresh weight.

In the field experiment, MgO NPs also improved fruit development and quality formation in mature fruiting trees. Fruit physical quality showed a clear concentration response (Figure 3a,b; Table S4). Single fruit weight increased with MgO NP concentration up to 200 mg/L, reaching 29.66 g, 70.38% higher than CK (Figure 3a). Fruit firmness was also enhanced by MgO NPs, with significant increases at 200–300 mg/L and a maximum increase of 56.25% over CK (Figure 3b). Fruit transverse and longitudinal diameters reached their highest values under 200 mg/L MgO NPs, increasing by 50.02% and 60.07%, respectively, whereas the fruit shape index was not significantly affected (Table S4). These results suggest that MgO NPs mainly promoted fruit enlargement and firmness rather than altering fruit shape. As suggested by previous studies, improved leaf photosynthetic pigment accumulation and mineral nutrient status may help increase assimilate supply to developing fruit, which could be associated with source-sink regulation during fruit expansion [34,35].

MgO NPs strongly improved loquat flavor quality by regulating soluble solids and acidity. Soluble solids content peaked at 14.57% under 200 mg/L MgO NPs, representing a 45.67% increase over CK (Figure 3c). In contrast, titratable acidity decreased to 0.31% at the same concentration, 53.26% lower than CK (Figure 3d). Consequently, the solid–acid ratio and sugar–acid ratio increased from 15.54 and 11.85 in CK to 47.96 and 40.28 under 200 mg/L MgO NPs, corresponding to increases of 208.62% and 240.06% (Figure 3e,f). At the equimolar Mg level, MgO NPs-50 treatment was more effective than MgSO_4_-50eq in increasing soluble solids and decreasing acidity (Figure 3c,d), suggesting that the improvement in flavor-related traits may not be solely attributable to Mg supply itself. The decline in flavor quality at higher concentrations indicates that the beneficial effect of MgO NPs on sugar–acid balance was weakened at excessive concentrations, again supporting the importance of dose control (Figure 3c–f). This interpretation is consistent with recent work showing that Mg application can regulate sugar–acid dynamics in loquat and other fruit crops through photosynthetic carbon supply, sugar transport, and organic acid metabolism [34,35,36].

### 2.4. MgO NPs Reshaped Soluble Sugar and Organic Acid Composition

**Figure 4:**
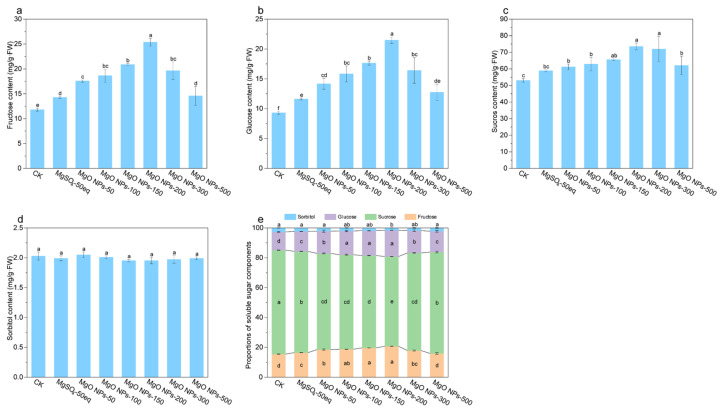
Effects of MgO NPs on the main components and contents of soluble sugars in loquat fruit. (**a**) Fructose content, (**b**) Glucose content, (**c**) Sucrose content, (**d**) Sorbitol content, (**e**) Proportions of soluble sugar components. Different lowercase letters above the bars indicate significant differences among different treatments (*p* < 0.05). Data are presented as mean ± SD, *n* = 3. FW stands for fresh weight.

The improvement in fruit flavor was further explained by changes in soluble sugar composition (Figure 4). MgO NPs significantly increased fructose, glucose, and sucrose contents, while sorbitol content was not significantly affected (Figure 4a–d). At 200 mg/L MgO NPs, fructose, glucose, and sucrose reached 25.38, 21.49, and 73.52 mg/g FW, increasing by 115.14%, 131.02%, and 38.53% over CK, respectively (Figure 4a–c). At the equimolar Mg level, MgO NPs-50 significantly increased fructose and glucose contents by 23.10% and 21.93% compared with MgSO_4_-50eq (Figure 4a,b). The proportional composition of soluble sugars also shifted: from 50 to 200 mg/L MgO NPs, the sucrose proportion gradually decreased, whereas fructose and glucose proportions increased (Figure 4e). At 200 mg/L, the sucrose proportion decreased by 9.55 percentage points compared with CK, while fructose and glucose proportions increased by 5.26 and 5.36 percentage points (Figure 4e). Because fructose and glucose are major sweetness-related sugars in loquat fruit, their increased contents and proportions likely contributed to the higher sugar–acid ratio. Similar Mg-mediated effects have been reported in fruit crops: foliar MgSO_4_ increased sucrose, fructose, and glucose contents in citrus by enhancing sucrose-metabolism enzymes and sugar-transporter genes [9], while appropriate Mg application improved soluble sugar accumulation, sugar–acid ratio, and sugar transport in ‘Red Fuji’ apple [35]. In loquat, 2% MgSO_4_ was also reported to increase soluble sugars and reduce malic acid content by 43.65%, thereby optimizing sugar–acid balance [34].

**Figure 5:**
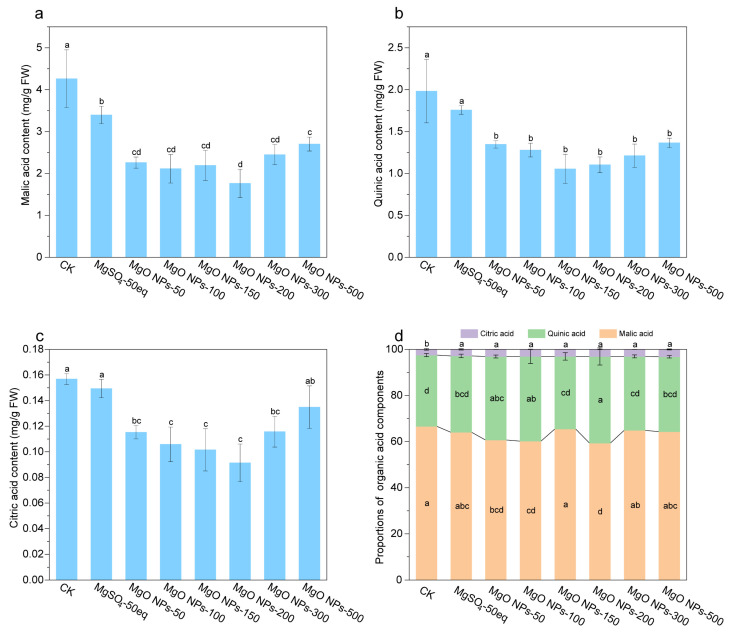
Effects of MgO NPs on the main components and contents of organic acids in loquat fruit. (**a**) Malic acid content, (**b**) Quinic acid content, (**c**) Citric acid content, (**d**) Proportions of organic acid components. Different lowercase letters above the bars indicate significant differences among different treatments (*p* < 0.05). Data are presented as mean ± SD, *n* = 3. FW stands for fresh weight.

Organic acid accumulation showed the opposite trend (Figure 5). All MgO NP treatments significantly reduced malic acid, quinic acid, and citric acid contents (Figure 5a–c). Quinic acid reached its lowest value at 150 mg/L MgO NPs (1.05 mg/g FW; 46.76% lower than CK; Figure 5b), whereas malic acid and citric acid reached their lowest values at 200 mg/L (1.76 and 0.09 mg/g FW; 58.59% and 47.14% lower than CK; Figure 5a,c). MgO NPs-50 also reduced all three organic acid components more strongly than MgSO_4_-50eq, with decreases of 33.43%, 23.28%, and 22.85%, respectively (Figure 5a–c). In the proportional profile, malic acid remained the dominant organic acid, followed by quinic acid and citric acid, but the malic acid proportion decreased by 7.28 percentage points at 200 mg/L MgO NPs (Figure 5d). These changes indicate that MgO NPs simultaneously enhanced sugar accumulation and reduced acid accumulation, producing a more favorable sugar–acid balance. Mg may contribute by activating enzymes involved in carbohydrate metabolism and sucrose cleavage, while also modulating organic acid biosynthesis and degradation pathways [34,36].

### 2.5. MgO NPs Enhanced Nutritional Metabolites and Altered Metabolism-Related Leaf Transcriptional Profiles

**Figure 6:**
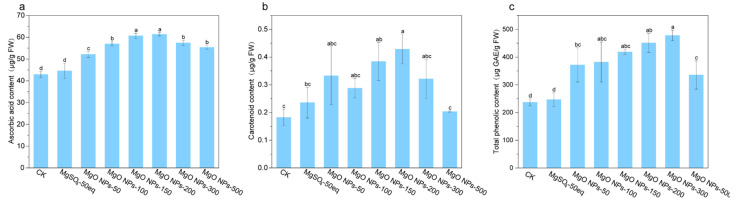
Effects of MgO NPs on contents of key metabolites in loquat fruit. (**a**) ascorbic acid content, (**b**) carotenoid content, (**c**) total phenol content. Different lowercase letters above the bars indicate significant differences among different treatments (*p* < 0.05). Data are presented as mean ± SD, *n* = 3. FW stands for fresh weight.

Fruit nutritional quality was also improved by MgO NP application (Figure 6). Ascorbic acid content increased to 62.34 μg/g FW under 200 mg/L MgO NPs, 45.26% higher than CK (Figure 6a). Carotenoid content also peaked at 200 mg/L, reaching 0.43 μg/g FW and increasing by 135.53% over CK (Figure 6b). Total phenolic content showed a slightly delayed optimum, increasing continuously up to 300 mg/L MgO NPs and reaching 478.11 μg GAE/g FW, 90.42% higher than CK (Figure 6c). Since ascorbic acid, carotenoids, and phenolics are important antioxidant metabolites in loquat fruit [2,3,4], their coordinated enhancement indicates that suitable MgO NP application can improve fruit nutritional quality by increasing antioxidant-related metabolites.

**Figure 7:**
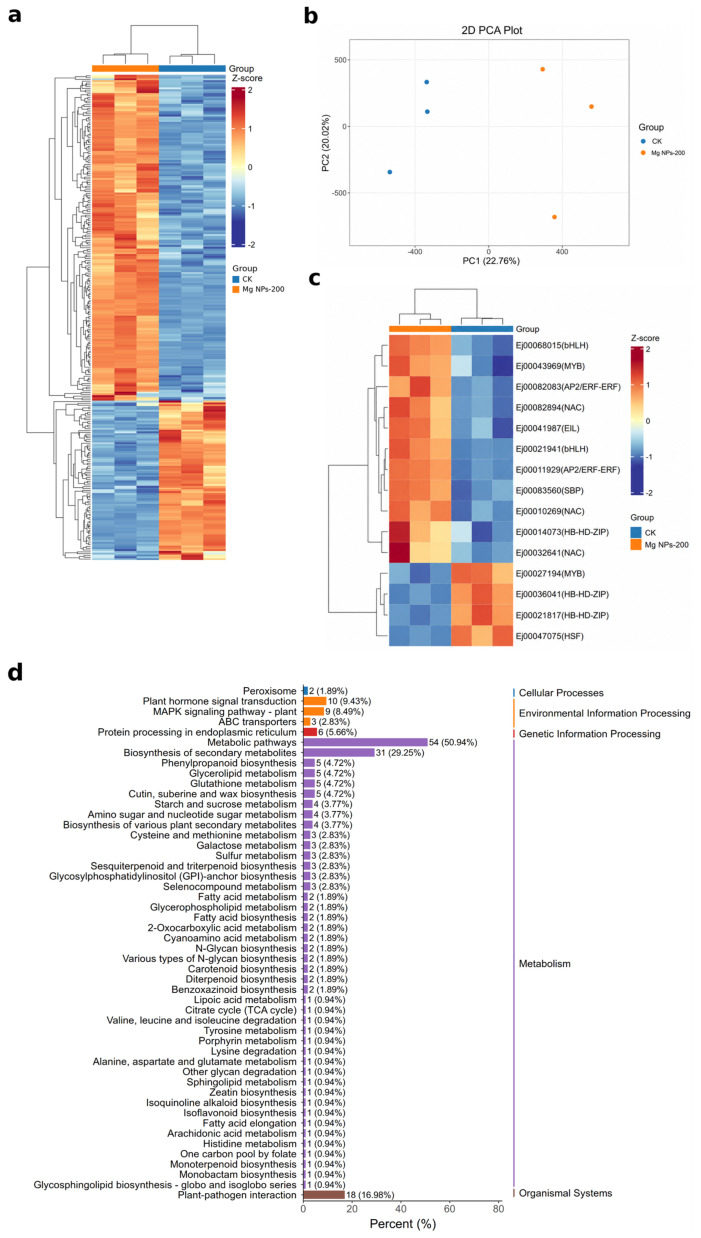
Transcriptome analysis of loquat leaves after foliar application of MgO NPs. (**a**) Hierarchical clustering heatmap of differentially expressed genes; (**b**) principal component analysis (PCA) plot; (**c**) clustering heatmap of differentially expressed transcription factors; (**d**) KEGG pathway classification of differentially expressed genes. CK represents the control, and MgO NPs-200 represents 200 mg/L MgO NP treatment.

To further explore the leaf transcriptional responses associated with MgO NP application, transcriptomic profiles of loquat leaves were analyzed. A total of 268 DEGs were identified between the MgO NPs-200 and CK treatments, including 180 upregulated and 88 downregulated genes. The hierarchical clustering heatmap of differentially expressed genes showed that CK and MgO NPs-200 samples clustered into two distinct groups, indicating that 200 mg/L MgO NPs markedly altered the gene expression pattern of loquat leaves (Figure 7a). PCA further showed partial separation between the two groups, with PC1 and PC2 explaining 22.76% and 20.02% of the variation, respectively, suggesting that MgO NPs induced changes in the transcriptomic profile of loquat leaves (Figure 7b).

The clustering heatmap of differentially expressed transcription factors showed that several members of the bHLH, MYB, AP2/ERF, NAC, EIL, and SBP families were up-regulated after MgO NP treatment, whereas some HB-HD-ZIP and HSF members were down-regulated (Figure 7c). AP2/ERF and NAC transcription factors are widely involved in plant stress responses, ethylene signaling, fruit ripening, and fruit quality regulation [37,38,39], while MYB and bHLH transcription factors commonly regulate phenylpropanoid and flavonoid biosynthesis [40]. Therefore, their differential expression suggests that MgO NPs may affect leaf transcriptional networks associated with stress responses, antioxidant metabolism, and secondary metabolism. The down-regulation of some HSF-related genes may also reflect changes in stress-response regulation, as HSF transcription factors are closely involved in heat stress responses, ROS homeostasis, and antioxidant defense in plants [41].

KEGG pathway analysis further showed that the DEGs were mainly assigned to metabolism-related pathways (Figure 7d). Among them, metabolic pathways contained the largest number of DEGs, with 54 genes accounting for 50.94%, followed by biosynthesis of secondary metabolites with 31 genes accounting for 29.25% (Figure 7d). In addition, plant-pathogen interaction, plant hormone signal transduction, and MAPK signaling pathway contained 18, 10, and 9 DEGs, respectively, suggesting that MgO NPs affected leaf genes associated with metabolic regulation and stress/signaling responses in loquat leaves. Several pathways related to metabolic regulation were also represented, including phenylpropanoid biosynthesis, glutathione metabolism, cutin, suberine and wax biosynthesis, starch and sucrose metabolism, amino sugar and nucleotide sugar metabolism, galactose metabolism, carotenoid biosynthesis, and glycerolipid metabolism (Figure 7d). The enrichment of DEGs in these pathways suggests that MgO NPs influenced leaf gene expression related to sugar metabolism, antioxidant defense, and secondary metabolism [40,42]. These leaf transcriptomic changes provide possible molecular clues for understanding the observed improvement in fruit quality.

## 3. Materials and Methods

### 3.1. Plant Cultivation and Experimental Soil

The test crop was loquat (*Eriobotrya japonica* Lindl.) cultivar ‘Ninghaibai’. The experimental soil was clayey red soil, classified as a red clay soil within the red soil subclass. Its parent material consisted of residual–slope deposits derived from the weathering of acidic igneous rocks, such as tuff. The soil was collected from a long-term loquat-growing area in Ninghai County, Ningbo City, Zhejiang Province, China (121.49° E, 29.21° N). The basic physicochemical properties of the soil are presented in Table S1. The exchangeable Mg content was 7.19 mg/kg, which was far below the critical threshold of 60 mg/kg, indicating severe Mg deficiency [6]. All chemicals and reagents used in this study were purchased from Aladdin Biochemical Technology Co., Ltd. (Shanghai, China), unless otherwise stated.

### 3.2. Characterization of MgO NPs

The physicochemical properties of the MgO NPs were characterized using multiple analytical techniques. Briefly, the MgO NP suspension was dropped onto a copper grid and air-dried prior to observation by transmission electron microscopy (TEM; JEM-1400, JEOL Ltd., Tokyo, Japan). The crystalline structure of the MgO NPs was determined by X-ray diffraction (XRD; D8 Advance, Bruker AXS GmbH, Karlsruhe, Germany) using Cu Kα radiation at 40 kV and 400 mA. The hydrodynamic diameter and zeta potential of the MgO NPs working suspension were measured by dynamic light scattering (DLS; Zetasizer Pro, Malvern Panalytical Ltd., Malvern, UK). As shown in Figure S1, the MgO NPs exhibited a cubic morphology and cubic-phase crystalline structure, with an average primary particle size of 52.6 ± 10.8 nm. The hydrodynamic diameter and zeta potential were 124.3 ± 31.0 nm and −34.0 ± 7.1 mV, respectively, indicating the nanoscale size and colloidal characteristics of the MgO NPs used in this study.

### 3.3. Experimental Design and Management

Pot and field experiments were conducted on loquat seedlings and mature fruit trees, respectively. The pot experiment was performed in a greenhouse at the West Zone of Zijingang Campus, Zhejiang University. Healthy and uniform one-year-old ‘Ninghaibai’ loquat seedlings were transplanted into pots containing 5 kg of sieved soil, which had been air-dried and passed through a 1 mm sieve. Surviving seedlings were selected for the experiment, with each seedling regarded as one experimental unit and three replicates per treatment. The field experiment was arranged in a randomized block design, with three replicate plots per treatment, four trees per replicate plot, and 2 m spacing between adjacent trees.

Two Mg fertilizers were applied by foliar spraying, and eight treatments were established for both pot and field experiments: (1) water control (CK), sprayed with water; (2) conventional Mg fertilizer comparator, MgSO_4_-50eq, 307.5 mg/L MgSO_4_·7H_2_O (1.25 mmol/L Mg), with the same molar Mg concentration as 50 mg/L MgO NPs; (3) MgO NPs-50, 50 mg/L (1.25 mmol/L Mg); (4) MgO NPs-100, 100 mg/L (2.5 mmol/L Mg); (5) MgO NPs-150, 150 mg/L (3.75 mmol/L Mg); (6) MgO NPs-200, 200 mg/L (5 mmol/L Mg); (7) MgO NPs-300, 300 mg/L (7.5 mmol/L Mg); and (8) MgO NPs-500, 500 mg/L (12.5 mmol/L Mg). The concentration settings were based on previous research [18,43]. The physicochemical characterization of MgO NPs is presented in Section 2.2 and Figure S1.

In the pot experiment, foliar spraying was conducted twice at 15-day intervals using a 1 L sprayer. Thirty days after the second application, leaf photosynthetic pigments, antioxidant enzyme activities, plant biomass, and total Mg, N, P, and K contents were determined. In the field experiment, foliar spraying was performed twice from the fruit expansion stage to the color-turning stage in mid-March, with a 15-day interval, using a 15 L electric sprayer. Spraying was conducted on sunny mornings before 10:00 a.m., and the treatment solution was applied evenly to both the adaxial and abaxial leaf surfaces until the leaves were uniformly wetted. Other agricultural practices were consistent with local farmer management. Leaves from the field experiment were collected 24 h after the first foliar application from fully expanded functional leaves at the middle canopy position, immediately frozen in liquid nitrogen, and stored at −80 °C for transcriptomic analysis. At fruit maturity, fruits were harvested directly from the trees. Fruits at similar commercial maturity and without visible disease, pest damage, or mechanical injury within each treatment were randomly collected from different canopy positions, with six fruits collected from each tree and 24 fruits obtained from each field replicate plot, preserved on dry ice, and transported to the laboratory as soon as possible for analysis. The mean value of the 24 fruits from each field replicate plot was used for subsequent statistical analysis. Some samples were used to determine fruit appearance quality, whereas the remaining samples were frozen in liquid nitrogen, ground, and stored at −80 °C for other fruit quality analyses.

### 3.4. Determination of Photosynthetic Pigment Contents

Chlorophyll a, chlorophyll b, and carotenoid contents were determined using a spectrophotometric method. Fresh mature leaves of loquat seedlings were collected, with the midribs removed, cut into pieces, and weighed to 0.1 g. The samples were placed in a mortar, mixed with a small amount of quartz sand and 5 mL of 80% acetone, and rapidly ground into a homogenate in an ice bath. Additional extraction solution was then added, and grinding was continued until the residue became completely white. The extract was filtered into a 25 mL brown volumetric flask. The mortar and residue were repeatedly rinsed with the solvent until the filter paper and residue showed no green color. The final volume was adjusted to the mark with the same extraction solution, mixed thoroughly, and extracted in the dark for 24 h. The absorbance was then measured at 663, 646, and 470 nm using a microplate reader (SPECTROstar Nano, BMG LABTECH, Ortenberg, Germany). All procedures were performed under dim light.

### 3.5. Antioxidant Enzyme Activities and Soluble Protein Content

The analysis was conducted using the methods described in previous reports [43]. Fresh mature leaves of loquat seedlings were collected, with the midribs removed, cut into pieces, and accurately weighed to 0.3 g. The samples were placed in a pre-cooled mortar, mixed with 3 mL of pre-cooled extraction buffer containing 50 mmol/L phosphate buffer (pH 7.8) and 1% polyvinylpyrrolidone, and rapidly ground into a homogenate in an ice bath. The homogenate was transferred into a centrifuge tube, and the mortar was rinsed twice with a total of 5 mL of extraction buffer. The rinsing solutions were combined with the homogenate. The mixture was centrifuged at 10,000–12,000 rpm for 20 min at 4 °C, and the supernatant was collected as the enzyme extract. Superoxide dismutase (SOD) activity was determined using the nitroblue tetrazolium (NBT) method, peroxidase (POD) activity using the guaiacol method, and catalase (CAT) activity using the ultraviolet absorption method. Soluble protein content was determined using the Coomassie Brilliant Blue G-250 method.

### 3.6. Elemental Content Analysis

The analysis was conducted using the methods described in previous reports [44]. Before drying, leaf samples were carefully rinsed with deionized water to remove surface-adhered residues from foliar spraying. Loquat seedlings were then heat-treated and dried at 80 °C to constant weight, then separated into roots, stems, and leaves. The samples were ground and passed through a 60-mesh (0.25 mm) nylon sieve. For total nitrogen (TN) determination, 0.2 g of sample was accurately weighed, digested using an H_2_SO_4_ + K_2_SO_4_/CuSO_4_ system, and analyzed by the Kjeldahl method. Another 0.2 g of sample was digested with an HNO_3_-H_2_O_2_ system using a DigiBlock digestion system (EH35A, LabTech, Rotherham, UK), and the contents of total phosphorus (TP), total potassium (TK), and total magnesium (TMg) were determined by ICP-OES (ICP 6000, Thermo Fisher Scientific, Waltham, MA, USA). All elemental concentrations were expressed on a dry-weight basis. For the calculation of biomass-based nutrient indices, element concentrations were uniformly expressed as mg/g DW before calculation. The accumulation and uptake indices of Mg, N, P, and K were calculated as follows:

Element accumulation amount (mg/plant) = organ dry biomass (g/plant) × element concentration (mg/g DW)

Element uptake amount (mg/plant) = element accumulation amount at the end of the experiment (mg/plant) − element accumulation amount at the beginning of the experiment (mg/plant)

Element uptake contribution rate (%) = [element uptake amount (mg/plant)/element accumulation amount at the end of the experiment (mg/plant)] × 100%

Element harvest index of a given organ (%) = [element accumulation amount in the organ (mg/plant)/total plant element accumulation amount (mg/plant)] × 100%

For the whole plant, total element accumulation was obtained by summing the accumulation amounts in roots, stems, and leaves. The same equations were used for Mg, N, P, and K.

### 3.7. Fruit Shape Index and Firmness

The analysis was conducted using the methods described in previous reports [44]. The longitudinal diameter, defined as the distance from the fruit apex to the fruit base (mm), and the transverse diameter, defined as the maximum diameter at the fruit equator (mm), were measured using a digital vernier caliper with an accuracy of 0.01 mm. Each fruit was measured at least twice, and the mean value was used. The fruit shape index was calculated as follows: fruit shape index = longitudinal diameter/transverse diameter. Fruit firmness was measured using a fruit firmness tester (GY-1, Beijing Penglichi Technology Co., Ltd., Beijing, China). Firmness was measured at five positions, including the fruit apex, two opposite points at the equator, and two symmetrical points at the fruit base, and the mean value was expressed as fruit firmness (kg/cm^2^).

### 3.8. Fruit Flavor Quality

The soluble solid content, soluble sugar components, titratable acid content, organic acid components, solid–acid ratio, and sugar–acid ratio of loquat fruits were determined [45,46]. The detailed methods are provided in Text S1.

### 3.9. Determination of Fruit Nutritional Quality

The ascorbic acid content, carotenoid content, and total phenol content of loquat fruits were determined [46,47,48]. The detailed methods are provided in Text S2.

### 3.10. Transcriptome Sequencing

Ribosomal RNA (rRNA) was removed from total RNA using the Ribo-Zero™ kit (Epicentre, Madison, WI, USA), and the enriched messenger RNA (mRNA) was used for transcriptome library construction. Each treatment included three biological replicates for RNA-seq. After library quality control, paired-end sequencing with a read length of 150 bp was performed on an Illumina NovaSeq 6000 high-throughput sequencing platform to generate raw sequence data. Raw sequencing data were preprocessed by quality filtering and adapter removal using fastp (version 0.23.2) to obtain high-quality clean reads for subsequent analysis. A total of 46.62 Gb of clean data was obtained, with at least 6 Gb of clean data for each sample. The percentage of Q30 bases was 96% or higher. Clean reads were mapped to the loquat reference genome using HISAT2 (version 2.2.1), with total mapping rates ranging from 94.47% to 96.17%. The mapped reads were assembled into transcripts using StringTie (version 2.1.6), and complete transcript information was obtained with reference to the loquat reference genome. Gene expression levels were calculated as FPKM (Fragments Per Kilobase of transcript per Million mapped reads) for expression description and visualization. Raw read counts were obtained using featureCounts (version 2.0.3), and differentially expressed genes (DEGs) were identified from raw count data using DESeq2 (version 1.22.1), with |log_2_(Fold Change)| ≥ 1 and FDR (False Discovery Rate) < 0.01 as the significance thresholds. Kyoto Encyclopedia of Genes and Genomes (KEGG) pathway enrichment analysis was then performed for the identified DEGs.

### 3.11. Statistical Analysis

The data were first processed using Excel and then statistically analyzed using SPSS 20.0 (IBM Corp., Armonk, NY, USA). Differences among treatments were analyzed by one-way analysis of variance (ANOVA), followed by Student–Newman–Keuls (S-N-K) post hoc tests at *p* < 0.05. All data are expressed as the mean ± standard deviation of replicate samples. Different lowercase letters indicate significant differences among treatments. Figures were generated using Origin 2026 (OriginLab Corporation, Northampton, MA, USA).

## 4. Conclusions

In conclusion, foliar application of MgO NPs improved loquat seedling growth, Mg nutrition, antioxidant-related physiological responses, and fruit quality under the present Mg-deficient acidic soil conditions. Among the tested concentrations, 200 mg/L MgO NPs showed the best overall performance, increasing seedling biomass, Mg uptake, soluble solids content, fruit size, sugar–acid balance, and nutritional metabolites, whereas 500 mg/L weakened several growth and quality responses. Based on the present field protocol, foliar application of 200 mg/L MgO NPs twice from the fruit expansion stage to the color-turning stage at a 15-day interval may serve as a preliminary practical reference for Mg supplementation in loquat orchards with Mg-deficient acidic soils. However, because this study was conducted under specific pot and field conditions, the long-term safety, economic feasibility, cost-effectiveness, and scalability of MgO NP application remain to be evaluated through multi-season and multi-site field trials before large-scale implementation.

## Data Availability

The original contributions presented in this study are included in the article/Supplementary Materials. Further inquiries can be directed to the corresponding author(s).

## Supplementary Materials

The following supporting information can be downloaded at: https://www.mdpi.com/article/10.3390/plants15132099/s1. Text S1: Determination of Fruit Flavor Quality; Text S2: Determination of Fruit Nutritional Quality; Table S1: Basic physicochemical properties of soil; Table S2: Effects of MgO NPs on the biomass of loquat seedlings; Table S3: Effects of MgO NPs on antioxidant enzyme activities and soluble protein content in loquat seedling leaves; Table S4: Effect of MgO NPs on fruit shape index of loquat fruit; Figure S1: Characterization of MgO NPs; Figure S2: Effects of MgO NPs on nitrogen accumulation amount, nitrogen uptake amount, nitrogen uptake contribution rate, and nitrogen harvest index of loquat seedlings; Figure S3: Effects of MgO NPs on phosphorus accumulation amount, phosphorus uptake amount, phosphorus uptake contribution rate, and phosphorus harvest index of loquat seedlings; Figure S4: Effects of MgO NPs on potassium accumulation amount, potassium uptake amount, potassium uptake contribution rate, and potassium harvest index of loquat seedlings; Figure S5: Correlation analysis of growth-related indicators in loquat seedlings [46,47,48].
