# Supplementary material for: Foliar Application of MgO Nanoparticles Modulates Magnesium Nutrition and Fruit Quality in Loquat Under Mg-Deficient Conditions
Source: Plants (Basel). 2026 Jul 6;15(13):2099. doi: 10.3390/plants15132099 (PMC13364114; doi:10.3390/plants15132099)
Supplement: Supplementary file 1 [file plants-15-02099-s001.zip › plants-4386668-supplementary.pdf]

Supporting Information

Foliar Application of MgO Nanoparticles Modulates Magnesium Nutrition and Fruit Quality in Loquat Under Mg-Deficient Conditions

Yuxiao Yang †, Jinrun Ni †, Wenkai Wang, Chang Lu, Jingjing Wan, Bilal Hussain, Xiaoe Yang * and Shane Wang *

State Key Laboratory of Soil Pollution Control and Safety, Zhejiang University, Hangzhou 310058, China

* Correspondence: xeyang@zju.edu.cn (X.Y.); shanewang@zju.edu.cn (S.W.); Tel./Fax: +86-0571-88982907 (X.Y.)

† These authors contributed equally to this work.

Text S1. Determination of Fruit Flavor Quality

Soluble solid content[1]

The soluble solid content of loquat pulp was determined using a handheld refractometer (WYT, Yancheng Leigu Technology Co., Ltd., China). Loquat pulp was cut into pieces and mixed, and the extracted juice was dropped onto the refractometer for measurement. Each treatment was measured in triplicate, and the mean value was calculated. The results were expressed as %.

Soluble sugar components[1]

The contents of soluble sugar components in loquat pulp were determined by high-performance liquid chromatography (HPLC). Different sugar components were qualitatively and quantitatively analyzed based on their retention times on the chromatographic column and detector responses.

Briefly, representative loquat pulp samples were frozen in liquid nitrogen, ground into fine powder, and stored at −80°C until analysis. A 1 g powder sample was accurately weighed, mixed with 5 mL of 80% ethanol, and ultrasonically extracted for 30 min at 40–50°C, with stirring two to three times during extraction. After centrifugation at 6,000–10,000 rpm for 15 min, the supernatant was passed through a C18 solid-phase extraction column to remove impurities such as pigments and phenolic compounds, and then filtered through a 0.22 μm microporous membrane to obtain the test solution.

A differential refractive index detector (1260 Infinity III, Agilent Technologies, USA) was used. Separation was performed on a Transgenomic COREGET-87C column (7.8

mm × 300 mm, 10 μm) at a column temperature of 85°C. Ultrapure water was used as the mobile phase at a flow rate of 0.6 mL/min. Fructose, sucrose, glucose, and sorbitol standard solutions with concentration gradients were analyzed under the same conditions. All standards were purchased from the National Sharing Platform for Reference Materials, China. Standard curves were established using concentration as the x-axis and peak area as the y-axis, and the contents of soluble sugar components were calculated accordingly. The results were expressed as mg/g FW, where FW indicates fresh weight. The calculation formulas were as follows:

$$C_i\text{（mg/g FW）} = \frac{c \times V}{m}$$

$$TSS = Fru + Suc + Glu + Sor$$

where C_i represents the content of a given soluble sugar component in the sample (mg/g FW), c represents the concentration obtained from the standard curve (mg/mL), V represents the final volume (mL), and m represents the fresh weight of the sample (g). TSS represents the total soluble sugar content (mg/g FW), Fru represents fructose content (mg/g FW), Suc represents sucrose content (mg/g FW), Glu represents glucose content (mg/g FW), and Sor represents sorbitol content (mg/g FW).

Titratable acid content[1]

The titratable acid content of loquat pulp was determined using the sodium hydroxide titration method. Briefly, 5 g of loquat pulp was placed in a mortar, mixed with a small amount of quartz sand, and rapidly ground into a homogenate. The homogenate was transferred into a 250 mL conical flask with

50 mL of distilled water and extracted in a constant-temperature water bath at 80°C for 30 min with continuous stirring. After cooling, the extract was filtered, and the residue was rinsed two to three times with a small amount of distilled water. The filtrates were combined and adjusted to a final volume of 100 mL with distilled water. Then, 20 mL of the test solution was mixed with two drops of phenolphthalein indicator and titrated with standard sodium hydroxide solution until a stable pink color appeared and did not fade, corresponding to pH 8.2. The volume of sodium hydroxide solution consumed was recorded, and the titratable acid content of the fruit samples was calculated. The results were expressed as the percentage (%) of malic acid equivalent using the following formula:

$$TA(\%) = \frac{c \times (V_1 - V_0) \times K \times F}{m} \times 1000$$

where TA represents the titratable acid content of the test solution (%), c represents the concentration of the standard sodium hydroxide solution (mol/L), V_1 represents the volume consumed for titrating the test solution (mL), V_0 represents the volume consumed for titrating the blank control (mL), K represents the conversion coefficient. Since the organic acids in loquat fruit are mainly malic acid, the K value was set as 0.067. F represents the dilution factor, m represents the fresh weight of the sample (g), and 1000 is the conversion coefficient.

Organic acid components[1]

The contents of organic acid components in loquat pulp were determined by

reversed-phase high-performance liquid chromatography (RP-HPLC). Representative loquat pulp samples were frozen in liquid nitrogen, ground into fine powder, and stored at −80°C until analysis. A 1 g powder sample was accurately weighed, mixed with 5 mL of 80% ethanol, and ultrasonically extracted for 30 min at 40–50°C, with stirring two to three times during extraction. After centrifugation at 6,000–10,000 rpm for 15 min, the supernatant was passed through a C18 solid-phase extraction column to remove impurities such as pigments and phenolic compounds, and then filtered through a 0.22 μm microporous membrane to obtain the test solution.

An ultraviolet detector was used for analysis. Separation was performed on a Waters μBondapak™ C18 column (3.9 mm × 300 mm, 10 μm) at a column temperature of 30°C. The mobile phase was 0.04 mol/L KH_2PO_4-H_3PO_4 buffer solution (pH 2.4), with a flow rate of 0.8 mL/min, and the detection wavelength was 214 nm. Malic acid, quinic acid, and citric acid standard solutions with concentration gradients were analyzed under the same conditions. All standards were purchased from the National Sharing Platform for Reference Materials, China. Standard curves were established using concentration as the x-axis and peak area as the y-axis, and the contents of organic acid components were calculated accordingly. The results were expressed as mg/g FW. The calculation formula was as follows:

$$C_i\text{（mg/g FW）} = \frac{C_s \times A_i \times V}{A_s \times m}$$

where C_i represents the content of a given organic acid in the sample (mg/g FW), C_s

represents the concentration of the organic acid standard solution (mg/mL), A_i represents the peak area of the sample, A_s represents the peak area of the standard, V represents the final volume (mL), and m represents the fresh weight of the sample (g).

Solid-acid ratio and sugar-acid ratio[2]

The solid-acid ratio and sugar-acid ratio of loquat fruit were calculated based on the measured soluble solid content, soluble sugar content, and titratable acid content.

Text S2. Determination of Fruit Nutritional Quality

Ascorbic acid[1]

The ascorbic acid content of loquat pulp was determined using the 2,6-dichloroindophenol titration method. Under acidic conditions, ascorbic acid reacts stoichiometrically with the dye, and the ascorbic acid content was calculated based on the consumption of the standard dye solution. The standard substance was purchased from the National Sharing Platform for Reference Materials, China.

Briefly, 5 g of loquat pulp was placed in a mortar, mixed with 5 mL of 2% oxalic acid and a small amount of quartz sand, and rapidly ground into a homogenate. The extract was filtered and transferred into a 50 mL volumetric flask. The residue was repeatedly rinsed with 2% oxalic acid, and the filtrates were combined and adjusted to 50 mL with 2% oxalic acid. Then, 10 mL of the filtrate was titrated with a blue alkaline 2,6-dichloroindophenol standard solution. During titration, 2,6-dichloroindophenol was reduced to a colorless form by ascorbic acid; at the endpoint, excess 2,6-dichloroindophenol appeared light red in the acidic medium. A 2% oxalic acid solution

was titrated in the same manner as the blank control. The ascorbic acid content was calculated according to the consumption of 2,6-dichloroindophenol and expressed as mg/kg FW. All procedures were performed under dim light, and grinding was completed as quickly as possible. The calculation formula was as follows:

$$AsA\ (\mu g/g\ FW) = \frac{(V - V_0) \times c}{m}$$

where AsA represents the ascorbic acid content (μg/g FW), V represents the dye volume consumed by the sample (mL), V_0 represents the dye volume consumed by the blank control (mL), c represents the mass concentration of the dye equivalent to ascorbic acid (mg/mL), and m represents the fresh weight of the sample (g).

Carotenoids[3]

The carotenoid content of loquat pulp was determined using a spectrophotometric method. Briefly, 0.5 g of loquat pulp was ground into powder in liquid nitrogen, mixed with 10 mL of acetone–petroleum ether (1:1, v/v), and extracted in the dark for 24 h. The mixture was shaken several times during extraction until the pulp became nearly colorless. After centrifugation at 8,000 rpm for 15 min at 4°C, the supernatant was collected for analysis. The extraction solvent was used as the blank control, and the absorbance was measured at 450 nm using a microplate reader (SPECTROstar Nano, BMG LABTECH, Germany). The total carotenoid content of loquat fruit was calculated and expressed as μg/g FW. The calculation was performed using the average molar absorption coefficient method according to the following formula:

$$Car\ (\mu g/g\,FW) = \frac{A_{450} \times V \times 10^4}{A_{1cm}^{1\%} \times m}$$

where Car represents the carotenoid content, A_{450} represents the measured absorbance at 450 nm, V represents the final volume (mL), $A^{1\%}{}_{1}cm$ represents the absorption coefficient of a 1% solution at a 1 cm optical path length, which was set as 2592 according to the absorption coefficient of β-carotene in petroleum ether, m represents the fresh weight of the sample (g), and 10^4 is the unit conversion factor.

Total phenols[1]

The total phenol content of loquat pulp was determined using the Folin–Ciocalteu colorimetric method. Under alkaline conditions, the Folin–Ciocalteu reagent is readily reduced by phenolic compounds, forming blue complexes consisting of molybdenum blue and tungsten blue. The color intensity is proportional to the phenolic content. Absorbance was measured at 765 nm, and the total phenol content was calculated using a gallic acid standard curve.

Briefly, 0.2 g of loquat pulp was mixed with 2 mL of methanol–formic acid extraction solution and ground in an ice bath. The homogenate was transferred into a centrifuge tube, and the residue was rinsed with a small amount of extraction solution and combined in the same tube. After ultrasonic extraction at 15°C for 30 min, the mixture was centrifuged at 8,000 rpm for 10 min at 15°C. Then, 100 μL of the supernatant was mixed with 1.5 mL of distilled water and 0.1 mL of Folin–Ciocalteu reagent. After mixing and standing for 1 min, 1.5 mL of 20% saturated sodium carbonate solution

was added. The mixture was thoroughly mixed and allowed to react in the dark for 2 h. The extraction solution was used as the blank control, and absorbance was measured at 765 nm using a microplate reader (SPECTROstar Nano, BMG LABTECH, Germany). Gallic acid was used as the standard, and the standard substance was purchased from the National Sharing Platform for Reference Materials, China. A standard curve was established, and the total phenol content was calculated accordingly. The results were expressed as gallic acid equivalents per fresh weight (μg GAE/g FW). The calculation formula was as follows:

$$TPC\text{（μg GAE/g FW）} = \frac{c \times V \times D}{m}$$

where TPC represents the total phenol content of the sample (μg GAE/g FW), c represents the concentration obtained from the standard curve (μg GAE/mL), V represents the final volume of the extract (mL), D represents the dilution factor, and m represents the fresh weight of the sample (g). GAE represents gallic acid equivalent.

Table S1. Basic physicochemical properties of soil.

Index	Value
pH	5.82 ± 0.20
SOM (g/kg)	27.95 ± 0.76
TN (g/kg)	2.03 ± 0.06
TP (g/kg)	1.58 ± 0.06
TK (g/kg)	18.31 ± 1.66
AHN (g/kg)	82.13 ± 1.05
AP (g/kg)	43.64 ± 0.98
AK (g/kg)	131.42 ± 9.40
Ex-Mg (g/kg)	7.19 ± 0.19

Note：Data are presented as mean ± SD, n=3.

Table S2. Effects of MgO NPs on the biomass of loquat seedlings.

Treat	Root	Above-ground	Stem	Leaf	Root-shoot ratio	Total
	g /plant DW	g /plant DW	g /plant DW	g /plant DW		g /plant DW
CK	0.81 ± 0.01^{d}	2.70 ± 0.06^{d}	1.25 ± 0.05^{c}	1.44 ± 0.02^{c}	0.301 ± 0.007^{d}	3.51 ± 0.06^{e}
$MgSO_4$-50eq	0.95 ± 0.04^{c}	3.03 ± 0.06^{c}	1.37 ± 0.05^{bc}	1.66 ± 0.02^{bc}	0.314 ± 0.009^{cd}	3.98 ± 0.10^{d}
MgO NPs-50	1.12 ± 0.09^{b}	3.35 ± 0.29^{b}	1.50 ± 0.16^{ab}	1.86 ± 0.13^{b}	0.335 ± 0.033^{bc}	4.47 ± 0.33^{c}
MgO NPs-100	1.35 ± 0.06^{a}	3.35 ± 0.01^{b}	1.48 ± 0.01^{ab}	1.86 ± 0.01^{b}	0.404 ± 0.019^{a}	4.70 ± 0.06^{bc}
MgO NPs-150	1.38 ± 0.05^{a}	3.48 ± 0.12^{b}	1.57 ± 0.04^{a}	1.91 ± 0.10^{b}	0.398 ± 0.027^{a}	4.86 ± 0.08^{ab}
MgO NPs-200	1.39 ± 0.15^{a}	3.77 ± 0.08^{a}	1.62 ± 0.05^{a}	2.15 ± 0.08^{a}	0.370 ± 0.038^{ab}	5.16 ± 0.20^{a}
MgO NPs-300	1.07 ± 0.07^{bc}	3.82 ± 0.11^{a}	1.63 ± 0.05^{a}	2.19 ± 0.09^{a}	0.280 ± 0.025^{cd}	4.89 ± 0.07^{ab}
MgO NPs-500	0.78 ± 0.02^{d}	2.98 ± 0.19^{d}	1.31 ± 0.11^{c}	1.67 ± 0.23^{bc}	0.263 ± 0.018^{d}	3.76 ± 0.19^{de}

Note： Different lowercase letters above indicate significant differences among different treatments ($p < 0.05$). Data are presented as mean ± SD, n=3. DW stands for dry weight. Same below.

Table S3. Effects of MgO NPs on antioxidant enzyme activities and soluble protein content in loquat seedling leaves.

Treat<br><br>mg/L	SOD activity<br><br>U/g FW	POD activity<br><br>U/g FW	CAT activity<br><br>U/g FW	Soluble protein content<br><br>mg/g FW
CK	99.49 ± 0.81^{c}	839.98 ± 15.01^{e}	5.19 ± 0.17^{b}	16.69 ± 0.61^{b}
$MgSO_4$-50eq	109.36 ± 7.78^{c}	865.50 ± 4.57^{d}	5.37 ± 0.31^{b}	17.92 ± 0.17^{b}
MgO NPs-50	134.03 ± 2.49^{b}	1030.14 ± 21.56^{c}	5.93 ± 0.25^{a}	19.79 ± 0.97^{a}
MgO NPs-100	141.41 ± 12.11^{b}	1132.73 ± 11.84^{b}	6.25 ± 0.44^{a}	19.82 ± 0.48^{a}
MgO NPs-150	146.61 ± 5.55^{ab}	1168.80 ± 9.37^{a}	6.17 ± 0.16^{a}	20.34 ± 0.57^{a}
MgO NPs-200	156.57 ± 5.85^{a}	1188.86 ± 17.16^{a}	6.33 ± 0.31^{a}	20.33 ± 0.63^{a}
MgO NPs-300	158.73 ± 5.40^{a}	1198.80 ± 16.00^{a}	6.41 ± 0.25^{a}	19.20 ± 0.93^{a}
MgO NPs-500	97.70 ± 3.23^{c}	877.40 ± 13.86^{d}	5.17 ± 0.17^{b}	16.63 ± 0.56^{b}

Note： Different lowercase letters above indicate significant differences among different treatments ($p < 0.05$). Data are presented as mean ± SD, n=3. FW stands for fresh weight. Same below.

Table S4. Effect of MgO NPs on fruit shape index of loquat fruit.

Treat mg/L	Transverse diameter mm	longitudinal diameter mm	Fruit shape index
CK	22.93 ± 1.19^{d}	24.47 ± 2.23^{c}	1.07 ± 0.09^{a}
$MgSO_4$-50eq	27.63 ± 0.83^{c}	31.37 ± 1.20^{b}	1.14 ± 0.06^{a}
MgO NPs-50	31.53 ± 2.04^{b}	34.30 ± 2.95^{ab}	1.09 ± 0.04^{a}
MgO NPs-100	31.17 ± 0.45^{b}	37.40 ± 0.17^{a}	1.20 ± 0.01^{a}
MgO NPs-150	33.10 ± 0.70^{ab}	38.27 ± 2.83^{a}	1.16 ± 0.07^{a}
MgO NPs-200	34.40 ± 1.90^{a}	39.17 ± 2.96^{a}	1.14 ± 0.07^{a}
MgO NPs-300	31.93 ± 1.01^{ab}	38.10 ± 2.65^{a}	1.19 ± 0.08^{a}
MgO NPs-500	30.37 ± 0.59^{b}	34.97 ± 1.94^{ab}	1.15 ± 0.06^{a}

Note: Different lowercase letters above indicate significant differences among different treatments ($p < 0.05$). Data are presented as mean ± SD, n=3.

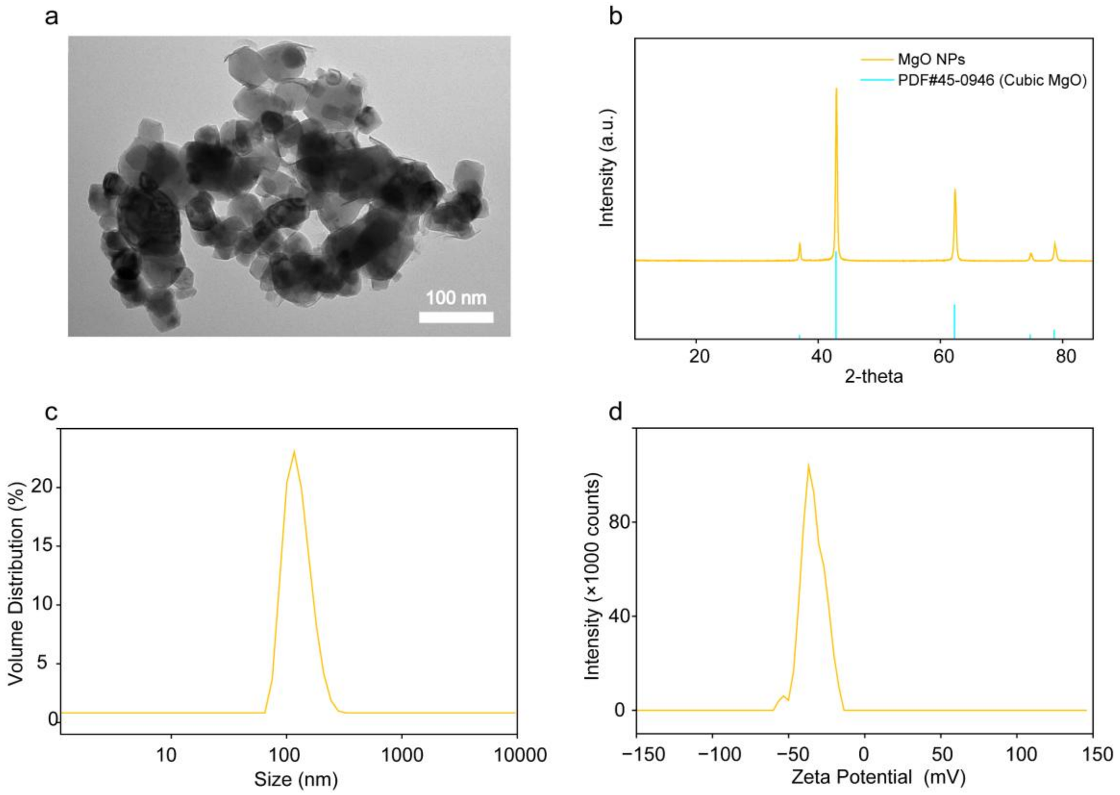

Figure S1. Characterization of MgO NPs. (**a**) TEM image showing that MgO NPs exhibited an irregular polyhedral morphology, with an average particle size of 52.6 ± 10.8 nm. (**b**) XRD pattern showing characteristic diffraction peaks at approximately 36.9°, 42.9°, 62.3°, 74.7°, and 78.6°, corresponding to the (111), (200), (220), (311), and (222) planes of cubic MgO, respectively, in agreement with the standard card PDF#45-0946. (**c**) DLS analysis showing a hydrodynamic diameter of 124.3 ± 31.0 nm in the working solution. (**d**) Zeta potential analysis showing a surface potential of −34.0 ± 7.1 mV, indicating moderate electrostatic repulsion and colloidal stability of MgO NPs.

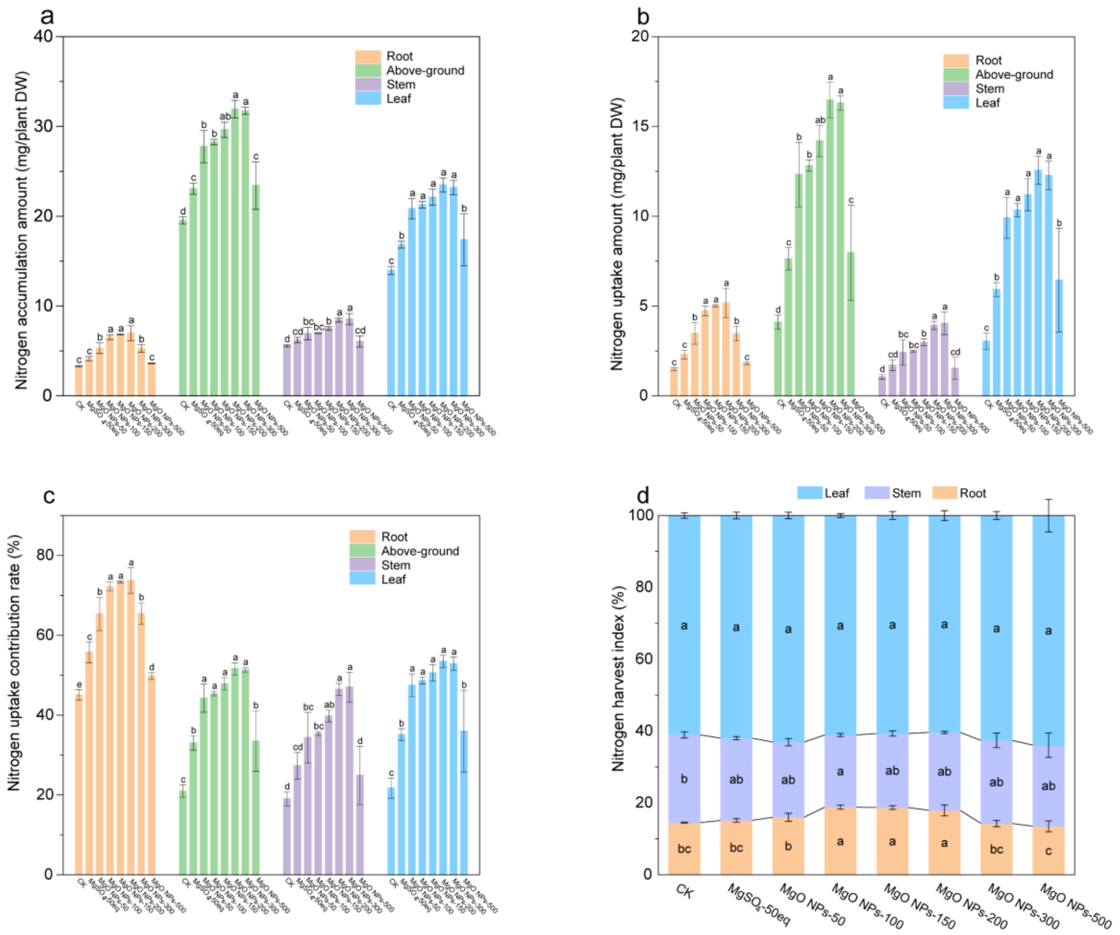

Figure S2. Effects of MgO NPs on (**a**) nitrogen accumulation amount, (**b**) nitrogen uptake amount, (**c**) nitrogen uptake contribution rate, and (**d**) nitrogen harvest index of loquat seedlings.

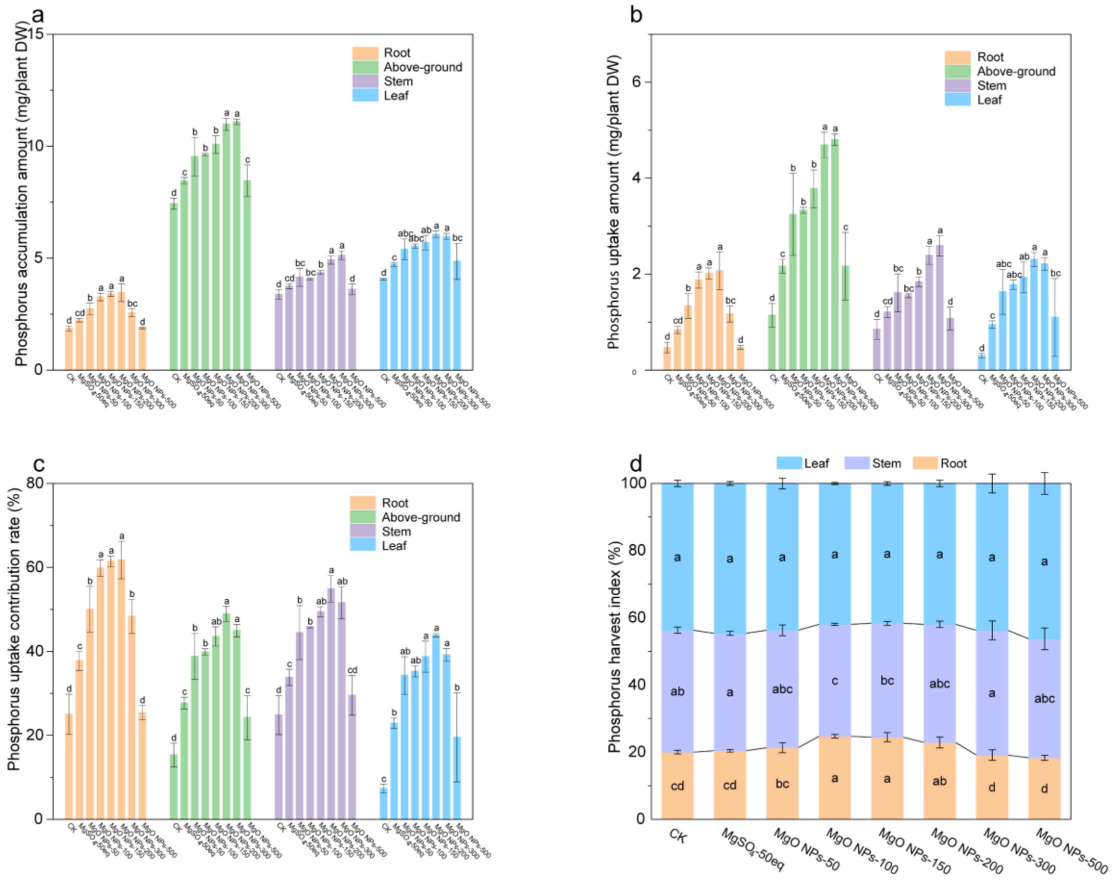

Figure S3. Effects of MgO NPs on (**a**) phosphorus accumulation amount, (**b**) phosphorus uptake amount, (**c**) phosphorus uptake contribution rate, and (**d**) phosphorus harvest index of loquat seedlings.

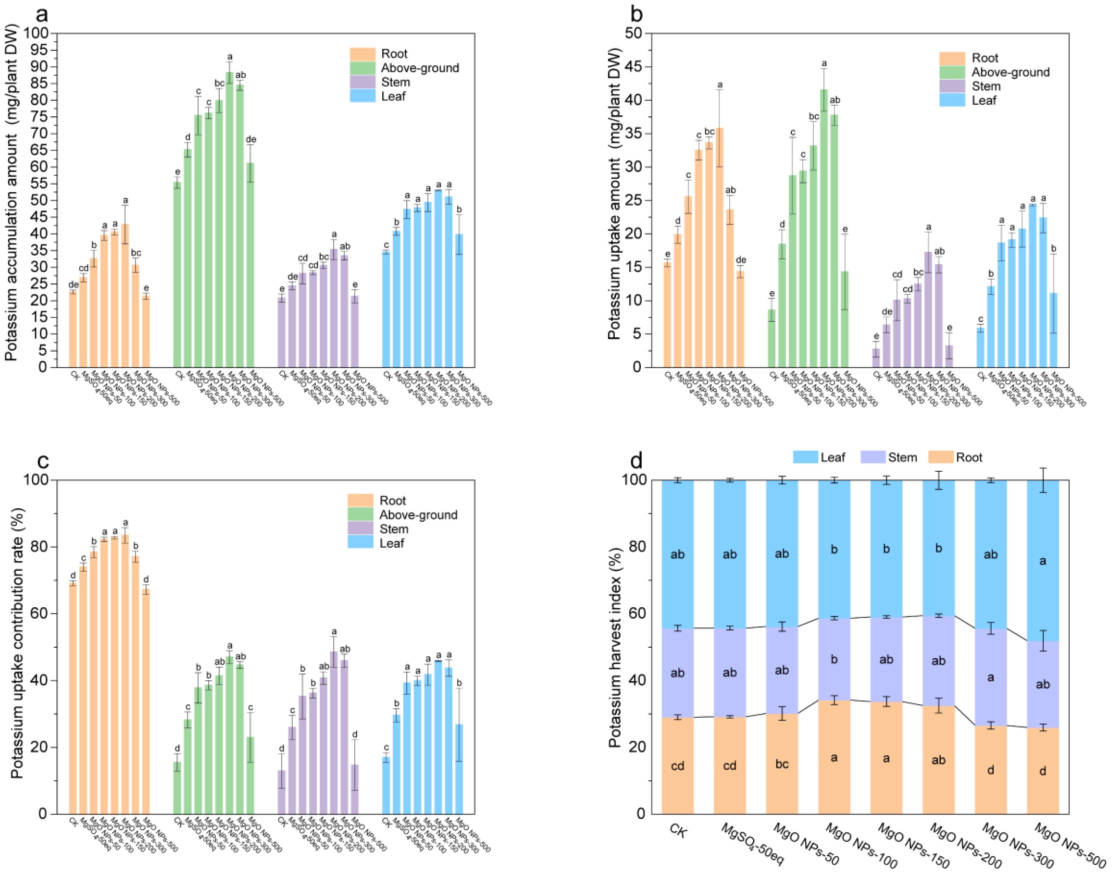

Figure S4. Effects of MgO NPs on (**a**) potassium accumulation amount, (**b**) potassium uptake amount, (**c**) potassium uptake contribution rate, and (**d**) potassium harvest index of loquat seedlings.

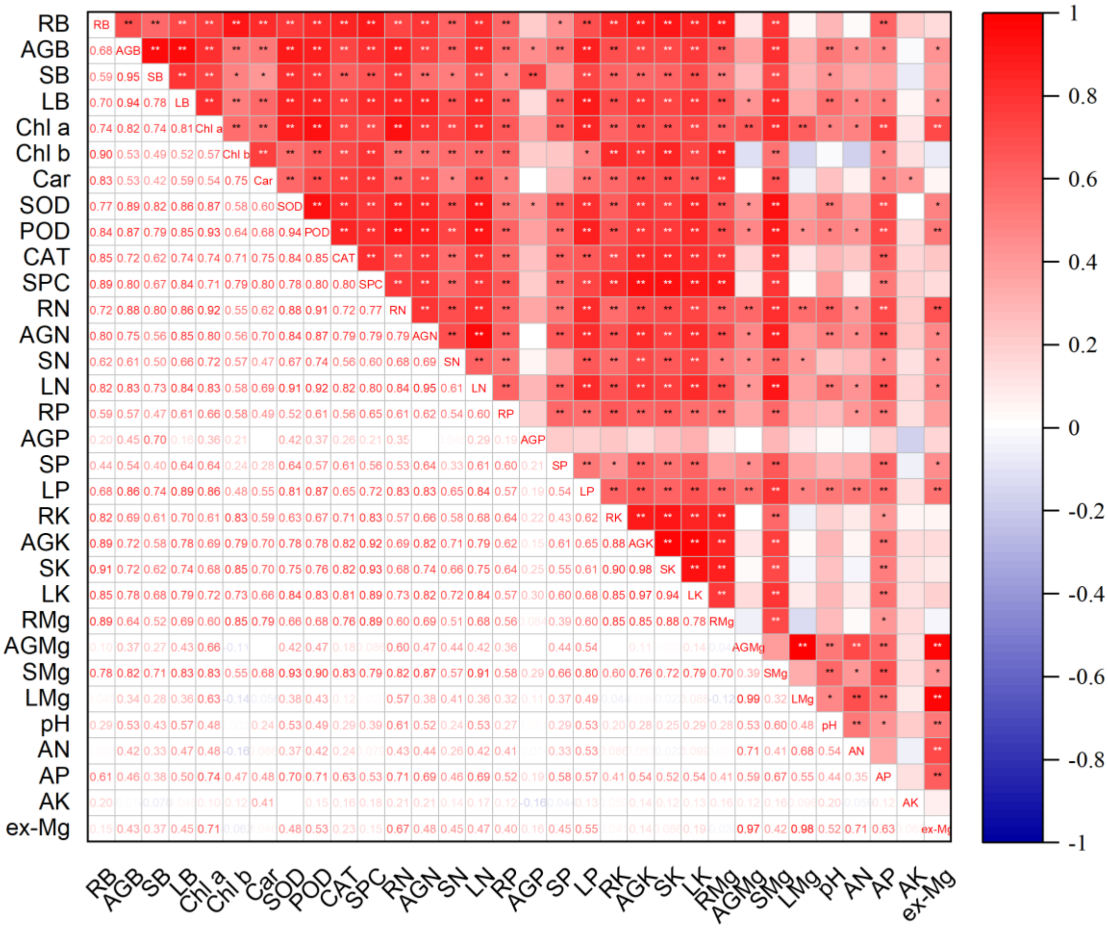

Figure S5. Correlation analysis of growth-related indicators in loquat seedlings. "*" indicates $p < 0.05$, "**" indicates $p < 0.01$. Abbreviations: RB = root biomass, AGB = above-ground biomass, SB = stem biomass, LB = leaf biomass, Chl a = chlorophyll a content, Chl b = chlorophyll b content, Car = carotenoid content, SOD = superoxide dismutase activity, POD = peroxidase activity, CAT = catalase activity, SPC = soluble protein content, RN = root total nitrogen content, AGN = above-ground total nitrogen content, SN = stem total nitrogen content, LN = leaf total nitrogen content, RP = root total phosphorus content, AGP = above-ground total phosphorus content, SP = stem total phosphorus content, LP = leaf total phosphorus content, RK = root total potassium content, AGK = above-ground total potassium content, SK = stem total potassium

content, LK = leaf total potassium content, RMg = root total magnesium content, AGMg = above-ground total magnesium content, SMg = stem total magnesium content, LMg = leaf total magnesium content, pH = soil pH, AN = alkaline hydrolyzable nitrogen content, AP = available phosphorus content, AK = available potassium content, ex-Mg = exchangeable magnesium content.

References:

1. Guirao, A.; Martínez-Romero, D.; Solana-Guilabert, A.; Agulló, V.; Díaz-Mula, H.M.; Valverde, J.M. Influence of Preharvest Sorbitol and Calcium-Sorbitol Applications on the Ripening Process and Anthocyanin Biosynthesis in Blood Orange (*Citrus Sinensis* Cv. Sanguinelli). *Food Chem.* **2025**, *481*, 144105, doi:10.1016/j.foodchem.2025.144105.
2. Prohaska, A.; Rey-Serra, P.; Petit, J.; Petit, A.; Perrotte, J.; Rothan, C.; Denoyes, B. Exploration of a European-Centered Strawberry Diversity Panel Provides Markers and Candidate Genes for the Control of Fruit Quality Traits. *Hortic. Res.* **2024**, *11*, uhae137, doi:10.1093/hr/uhae137.
3. Dhuique-Mayer, C.; Fanciullino, A.-L.; Dubois, C.; Ollitrault, P. Effect of Genotype and Environment on Citrus Juice Carotenoid Content. *J. Agric. Food Chem.* **2009**, *57*, 9160–9168, doi:10.1021/jf901668d.
